# Evidence Drift and Causal Maturation Drift in Pediatric Health Research: A Dual-Drift Framework and Preliminary Appraisal Instruments for Inferential Fidelity

**DOI:** 10.3390/children13091161

**Published:** 2026-08-28

**Authors:** Ziad D. Baghdadi

**Affiliations:** Independent Researcher, Winnipeg, MB R3Y1P5, Canada; drziadeddin.albaghdadi@gmail.com or zalbaghdadi@atsu.edu

**Keywords:** evidence drift, causal maturation drift, inferential fidelity, causal inference, translational research, research waste, meta-research, early childhood caries, pediatric research, child health

## Abstract

**Highlights:**

**What are the main findings?**

- **Two distinct modes of research failure are identified:** Scientific progress in pediatric health research can fail either through inferential overextension or developmental under-resolution.
- **Evidence Drift (ED) is formalized:** This is a claim-level failure in which research conclusions shift to a stronger or different inferential domain without an adequate evidentiary bridge.
- **Causal Maturation Drift (CMD) is formalized:** This is a trajectory-level failure in which a field continues to publish within an established domain, even though the principal decision-relevant uncertainty remains unaddressed.
- **The Dual-Drift Framework is established: ** Governed by the principle of *Inferential Fidelity*, the framework requires alignment among the research question, design, claim, remaining uncertainty, the next study, and implementation safeguards
- **Structured appraisal instruments are introduced:** Two preliminary eight-item scales—the Evidence Drift Assessment Scale (EDAS) and the Causal Maturation Drift Assessment Scale (CMDAS—are proposed for empirical development.

**What are the implications of the main findings?**

- **Structured evaluation for stakeholders:** The framework equips investigators, reviewers, funders, guideline panels, and policymakers with a systematic approach for determining whether conclusions remain within their evidentiary boundaries.
- **Strategic portfolio management:** It allows stakeholders to assess whether ongoing research activity is actively reducing the uncertainties that matter most to children, families, and clinical decisions, rather than merely accumulating lateral publications.
- **Safeguarding pediatric evidence:** By distinguishing claim appraisal from trajectory appraisal, the framework helps prevent the premature substitution of surrogate outcomes, short-term endpoints, or adult data for evidence of developmentally appropriate benefit in pediatric care.

**Abstract:**

Pediatric health research must translate evidence into decisions that affect children’s development, safety, function, and long-term well-being. Scientific progress can still fail in two opposing ways: claims may exceed their evidentiary support, or research programs may remain productive while repeatedly refining an established signal without resolving decision-relevant uncertainty. These risks are amplified by developmental heterogeneity, ethical constraints, surrogate or short-term outcomes, long follow-up, and caregiver-mediated implementation. This concept paper formalizes these failures as Evidence Drift (ED) and Causal Maturation Drift (CMD). ED is a claim-level failure in which a finding moves into a stronger or different inferential domain without an adequate bridge. CMD is a trajectory-level failure in which research continues to accumulate within an established domain after a signal is sufficiently characterized, without proportionate progression toward temporal, causal, comparative, long-term, or implementation evidence. Inferential Fidelity is proposed as the governing principle linking claim calibration to purposeful uncertainty reduction. Applications are illustrated through early childhood caries microbiome research, vitamin D and childhood caries, silver diamine fluoride, pediatric biomarker and omics pipelines, and artificial intelligence prediction studies. Two preliminary eight-item appraisal frameworks are introduced: the Evidence Drift Assessment Scale (EDAS) for claims and the Causal Maturation Drift Assessment Scale (CMDAS) for literature trajectories. These formative frameworks prioritize item-level profiles; any standardized index is secondary and non-diagnostic. A phased validation program includes content validation, cognitive testing, multi-assessor reliability, hypothesis-based construct testing, bibliometric trajectory mapping, and evaluation of practical utility. The framework offers investigators, reviewers, funders, guideline panels, and policymakers a structured approach to determining whether conclusions remain within evidentiary boundaries and whether research activity reduces the uncertainties that matter most to children and families. Transferability beyond pediatric research requires empirical testing.

## 1. Introduction

In pediatric health research, translational frameworks describe how observations on childhood exposures, development, disease, prevention, and treatment can move from discovery to clinical application, evidence-based recommendations, implementation, and population benefit [1,2]. These pathways are neither automatic nor uniformly linear. Reviews of translational time lags show substantial variation in what is translated, how progression is measured, and the duration of different pathways [3]. The difficulty is not simply that science moves slowly. More fundamentally, as highlighted by meta-research, scientific activity may be poorly aligned with the unresolved inferential and clinical questions.

Research-waste and meta-research studies have documented preventable losses stemming from low-priority questions, avoidable design limitations, selective analysis, incomplete reporting, and weak placement of new studies within the existing evidence base [4,5,6,7,8]. Work on spin and inappropriate extrapolation has shown that statistically or clinically limited findings may be presented more favorably than warranted, while the surrogate-endpoint literature has long warned that improvement in an intermediate outcome does not automatically establish patient benefit [9,10,11]. These contributions identify major failures in the production and communication of evidence.

These concerns are particularly important in pediatric health research. Developmental stage can modify exposures, mechanisms, treatment effects, outcome measurement, and the consequences of error. Ethical and practical constraints may limit experimentation, while relatively small or heterogeneous samples, reliance on caregiver reports or surrogate outcomes, and the need for long-term follow-up may make observational and short-term evidence especially prominent. Implementation is also frequently mediated by caregivers, schools, community services, and health systems. These characteristics do not diminish pediatric evidence; rather, they underscore the importance of maintaining proportionality among the question, design, results, claims, and the next research step.

Despite these contributions, an important analytical gap remains. Existing frameworks do not fully distinguish between two opposing directional errors: overextending a claim beyond its supporting inferential domain and failing to advance a research trajectory toward reducing uncertainty despite continued publication. The first is a claim-level failure; the second is a trajectory-level failure. Distinguishing them is important because they require different units of analysis, different evidence for appraisal, and different corrective responses. As summarized in Table 1, the Dual-Drift Framework addresses a distinct analytical gap compared with existing meta-research concepts:

- Spin focuses primarily on favorable framing or selective emphasis within a publication, but it does not determine whether a claim has crossed into an unsupported inferential domain (e.g., from statistical association to causation).
- Research waste identifies broad losses from low-value questions or weak methods, but it does not isolate a technically sound research trajectory that repeatedly refines a lower-priority question while leaving core uncertainties unresolved.
- Translational delay concerns the time required for mature evidence to reach practice, whereas Causal Maturation Drift concerns whether the evidence itself is progressing toward decision readiness (i.e., having the necessary causal, comparative, and implementation support to safely inform clinical or policy action).

Evidence Drift and Causal Maturation Drift therefore do not replace these established concepts; they add a directional, unit-specific framework linking claim calibration with the maturation of research trajectories.

To address this gap, this paper formalizes two complementary concepts—Evidence Drift and Causal Maturation Drift—unified by the governing principle of Inferential Fidelity. A previously published field-specific article applied Evidence Drift to early childhood caries (ECC) within a causal-translation framework—a model that maps how etiological findings progress through requisite evidentiary bridges into clinical and population interventions [12]. The present manuscript significantly extends that work by (1) formalizing Causal Maturation Drift as a general trajectory-level construct; (2) establishing the two-axis Dual-Drift Model; (3) broadening applications across pediatric etiological, clinical, technological, and implementation research; and (4) proposing preliminary, structured appraisal domains (EDAS and CMDAS) for empirical development.

Evidence Drift is an inferential error: an association may be described as causal; a plausible mechanism may be treated as proof of effectiveness; a short-term therapeutic outcome may be presented as disease resolution; or uptake may be equated with equitable implementation. Causal Maturation Drift is a developmental error: a field may generate additional cohorts, molecular profiles, predictive models, or short-term intervention estimates after the original signal has become sufficiently visible, yet devote comparatively little effort to the temporal, experimental, comparative, or implementation questions needed to reduce the most important remaining uncertainty.

The objectives are therefore to (1) provide formal definitions and boundary conditions for pediatric health research; (2) distinguish the constructs from spin, research waste, replication, and translational delay; (3) illustrate how the two drifts may occur independently or together across pediatric etiological, clinical, and implementation research; (4) conceptually propose preliminary claim- and trajectory-level scales for future empirical development in the structured appraisal of pediatric evidence; and (5) outline a validation and implementation agenda, including the empirical prerequisites for future testing of scale transferability beyond pediatrics.

A complete Glossary of Key Terms and definitions for all Dual-Drift constructs is provided at the end of the manuscript for quick reference.

## 2. Conceptual Positioning

### 2.1. Scientific Maturation Is Uncertainty Reduction, Not a Fixed Sequence

Causal inference requires explicit attention to temporality, confounding, selection, measurement, intervention contrasts, and the assumptions linking data to causal claims [13,14,15]. Triangulation strengthens inference when approaches with different, preferably unrelated, sources of bias address the same question [16,17]. Yet no universal study sequence is appropriate for every field. Ethical constraints may preclude randomization; rare outcomes may require observational designs; mechanisms may be investigated before or after population effects are observed; and implementation findings may generate reverse-translation questions.

In pediatric research, appropriate maturation may require age-stratified longitudinal follow-up, developmentally valid outcomes, and methods that account for family, school, community, and health-system contexts. The absence of randomized evidence should not, in itself, be interpreted as immaturity; the relevant question is whether the chosen design reduces the most consequential uncertainty while remaining within legitimate ethical and developmental constraints.

Accordingly, maturation is defined here functionally rather than hierarchically: a research trajectory matures when successive studies reduce consequential uncertainty, clarify the conditions under which a claim is valid, or demonstrate that a hypothesis should be narrowed, redirected, or abandoned. Confirmation is not required. A null Mendelian randomization analysis, a trial that weakens a plausible intervention claim, or an implementation study that reveals unacceptable inequity can all signal maturation because each improves decision-relevant understanding.

### 2.2. Different Units of Analysis

Evidence Drift is primarily assessed at the level of a claim: the conclusion of a study, the interpretation in a review, a recommendation in a guideline, or a policy statement. It may be identifiable within a single document. Causal Maturation Drift is assessed at the level of a trajectory: a body of literature, a research program, a technology pipeline, or a funding portfolio over time. An individual study may contribute to the pattern, but one study alone cannot establish it. Table 1 distinguishes the Dual-Drift Framework from adjacent meta-research concepts by comparing their primary units of analysis, principal questions, evidentiary requirements, and unique analytical contributions.

**Table 1:** Distinguishing the Dual-Drift Framework from Adjacent Meta-Research Concepts.

Concept	Primary Unit of Analysis	Principal Question Asked	Evidence Required for Assessment	Unique Analytical Feature Added
Evidence Drift (ED)	Claim, review recommendation, or policy statement	*Has the conclusion crossed into a stronger or different inferential domain without an adequate bridge?*	Single study, synthesis, or guideline document plus domain mapping	Evaluates directional inferential overextension across domains (e.g., association → causation; short-term endpoint → long-term resolution)
Causal Maturation Drift (CMD)	Literature trajectory, research program, or funding portfolio	*Does persistent publication continue within an established domain without reducing principal residual uncertainty?*	Mapped literature trajectory over time, cumulative review, or portfolio data	Measures lateral accumulation vs. uncertainty-reducing progression over time; isolates productive stagnation
Spin	Report, abstract, or news release	*Is the presentation framed more favorably than the results warrant?*	Study text and reported results	Addresses rhetoric and selective emphasis within a report, regardless of whether domain boundaries are crossed [9,10]
Research Waste	Study, research enterprise, or system	*Has value been lost through bad questions, poor design, non-publication, or unusable reporting?*	Methodological evaluation of design, conduct, and reporting	Identifies broad inefficiency; CMD specifically highlights cases where *technically sound* studies accumulate on lower-priority uncertainties [4,5,6,7,8]
Translational Delay/Gap	Discovery-to-practice pathway	*Why has validated evidence taken extended time to reach clinical implementation?*	Longitudinal tracking of benchmark milestones over elapsed time	Focuses on elapsed time for *mature* evidence; CMD evaluates whether the evidence *itself* is maturing toward decision readiness [1,2,3]
Replication	Study or body of studies	*Is the observed effect size robust across independent samples or settings?*	Independent studies testing the same hypothesis/design	Necessary replication strengthens signal stability; CMD begins *after* a signal is sufficiently characterized for the decision at hand
GRADE certainty and indirectness	Body of evidence for an outcome or recommendation	*How certain is the estimated effect, considering risk of bias, inconsistency, indirectness, imprecision, and publication bias?*	Systematic evidence profile and certainty assessment	GRADE evaluates certainty in an effect estimate; ED evaluates whether a claim crosses an inferential domain, while CMD evaluates whether a trajectory progresses toward resolving the decision-relevant uncertainty.
Value-of-information analysis	Decision problem and candidate research investment	*Would additional research improve expected decisions sufficiently to justify its cost?*	Probabilistic decision model and economic analysis	Value-of-information methods quantify the expected return from additional research; CMD provides a qualitative diagnostic of under-resolution, repeated limitations, and missing evidentiary bridges, including when formal economic modeling is unavailable.

### 2.3. Concept-Development Approach and Evidentiary Status

This paper used iterative conceptual synthesis rather than a systematic review or formal consensus process. Established literature on causal inference, spin, surrogate endpoints, research waste, replication, translational research, and implementation was compared by direction of error and unit of analysis. Candidate constructs and domains were refined by assessing whether they consistently distinguished claim-level overextension from trajectory-level under-resolution across pediatric etiological, clinical, biomarker, technological, and implementation examples. Examples were selected purposively to examine conceptual breadth and boundary conditions, not to estimate prevalence or classify research fields. Evidence Drift, Causal Maturation Drift, EDAS, and CMDAS should therefore be regarded as proposed constructs and appraisal domains requiring independent content validation, reliability testing, and empirical refinement. The proposed domains were retained when they represented conceptually distinct failure modes, applied across more than one pediatric research context, and generated a corresponding corrective response. This process was interpretive rather than exhaustive; no claim is made that the proposed domains capture every form of inferential overextension or trajectory-level under-resolution.

## 3. Evidence Drift: Inferential Overextension

**Evidence Drift (ED)** is the movement of a finding toward a stronger or different causal, clinical, implementation, or policy claim without an adequate evidentiary bridge.

Crossing domains is not inherently problematic. Translation is essential. Drift occurs when the bridge is missing, inadequate, or hidden. Appropriate bridges may include temporal evidence, causal identification, experimental manipulation, a validated linkage between surrogate and patient-important outcomes, external validation, comparative effectiveness, implementation evaluation, or explicit retention of uncertainty and context.

Several recurring forms can be distinguished. Causal escalation occurs when an association is described as causal without an identification strategy. Mechanistic substitution occurs when biological plausibility is treated as proof that modifying the mechanism improves outcomes. Endpoint inflation occurs when an intermediate, short-term, or lesion-level outcome is expanded into a claim of disease resolution or comprehensive benefit. Contextual extrapolation occurs when findings from one population, setting, exposure range, or follow-up period are generalized without support. Implementation overreach occurs when efficacy, uptake, or reach is presented as proof of real-world effectiveness, sustainability, safety, or equity. These forms overlap with known categories of spin and surrogate misuse but are organized around the specific question of whether the conclusion has crossed an inferential boundary [9,10,11].

In pediatric research, contextual extrapolation also includes directly applying adult evidence to children, combining developmentally distinct age groups without justification, or assuming that short-term effects will persist through growth and maturation. Endpoint inflation may similarly occur when a biomarker, lesion-level change, caregiver-reported process measure, or brief symptom improvement is presented as evidence of a durable child health benefit.

Evidence Drift can occur even when the underlying study is rigorous. A well-conducted cross-sectional study may support a robust association, yet a causal conclusion would still be inappropriate. A randomized trial may establish an effect on a narrow endpoint, yet a recommendation for long-term comprehensive care may still exceed the scope of the tested outcome. The construct therefore evaluates claim calibration rather than ranking study designs.

## 4. Causal Maturation Drift: Developmental Under-Resolution

**Causal Maturation Drift** is a persistent trajectory-level pattern in which a field continues to generate, replicate, or refine associative findings or narrow-domain outcomes after a signal has been sufficiently characterized, without proportional progress toward the temporal, mechanistic, causal, comparative, long-term, or implementation evidence needed to resolve the principal decision-relevant uncertainty.

The defining movement is lateral rather than static. Publications, datasets, technologies, and analytic detail may increase, while the central question changes little. More precise descriptions of the same association, new subgroups using the same design, additional molecular signatures measured after disease onset, or repeated short-horizon estimates of the same therapeutic endpoint may all yield legitimate incremental knowledge. CMD is suspected only when these increments become persistently disproportionate to the unresolved bridge that matters most for causal understanding or patient care.

Four conditions should be considered before assigning the label. First, the trajectory and time window must be defined. Second, there must be a defensible basis for judging that the original signal is sufficiently characterized for the intended decision; this may rely on replication, systematic review, cumulative meta-analysis, or expert consensus. Third, the core uncertainty must be stated explicitly. Fourth, the lack of progression must not be adequately explained by scientific, ethical, feasibility, or resource constraints. CMD is therefore not a criticism of early discovery, necessary replication, understudied populations, or methodologically justified refinement.

Several manifestations are possible. Associative saturation refers to repeated exposure–outcome studies conducted after the signal has already stabilized. Mechanistic recursion refers to increasingly detailed laboratory or omics explanations that lack sufficient linkage to temporality or intervention. Technology-led refinement occurs when a new platform generates more granular versions of an established observation but does not answer the next clinical question. Endpoint confinement occurs when a therapeutic field repeatedly studies a narrow or short-term outcome while patient-important, comparative, or long-term outcomes remain underdeveloped. Implementation deferral occurs when evidence of efficacy continues to expand but delivery, safety, sustainability, and equity are repeatedly postponed.

In child health research, endpoint confinement may be especially consequential when studies repeatedly measure biomarkers, technical performance, lesion status, or short-term symptoms, while growth, development, function, participation, quality of life, family burden, long-term safety, and continuity of care remain underexamined.

The term *causal* is used deliberately and extends beyond etiological identification. Here, causal maturation encompasses the full decision-relevant chain linking an exposure or intervention to outcomes under specified conditions: what changes when the exposure or intervention is modified, for whom, over what period, against which alternative, and within which delivery context. CMD may therefore arise both upstream, when etiological evidence fails to move beyond association, and downstream, when disease-control evidence fails to mature into comparative, long-term, and implementation knowledge.

Maturation should be judged by information gain rather than by confirmation. A research trajectory may mature by supporting a hypothesis, narrowing it to a subgroup, revealing effect modification, showing that a proposed mechanism is not clinically important, or determining that further investment is unjustified. The opposite of CMD is not a positive result; it is a disciplined reduction in the uncertainty that matters.

## 5. Inferential Fidelity and the Dual-Drift Model

**Inferential Fidelity** is the preservation of alignment among the question posed, the design used, the result obtained, the claim advanced, the uncertainty that remains, the next research step selected, and the safeguards maintained throughout implementation.

Inferential Fidelity has four linked dimensions. Question-design fidelity asks whether the method can answer the stated question. Result-claim fidelity asks whether the conclusion is commensurate with the evidence. Uncertainty-next-study fidelity asks whether subsequent research targets the most consequential unresolved issue rather than merely reproducing the accessible one. Evidence-implementation fidelity asks whether real-world delivery preserves diagnosis, monitoring, safety, referral, patient-centered outcomes, and equity, when relevant.

In pediatric settings, Evidence-Implementation Fidelity also requires developmentally appropriate diagnosis and outcome measurement, appropriate consent and assent processes, meaningful caregiver involvement, safeguarding, age-appropriate monitoring, and attention to inequities affecting access, referral, and follow-up.

**Figure 1:**
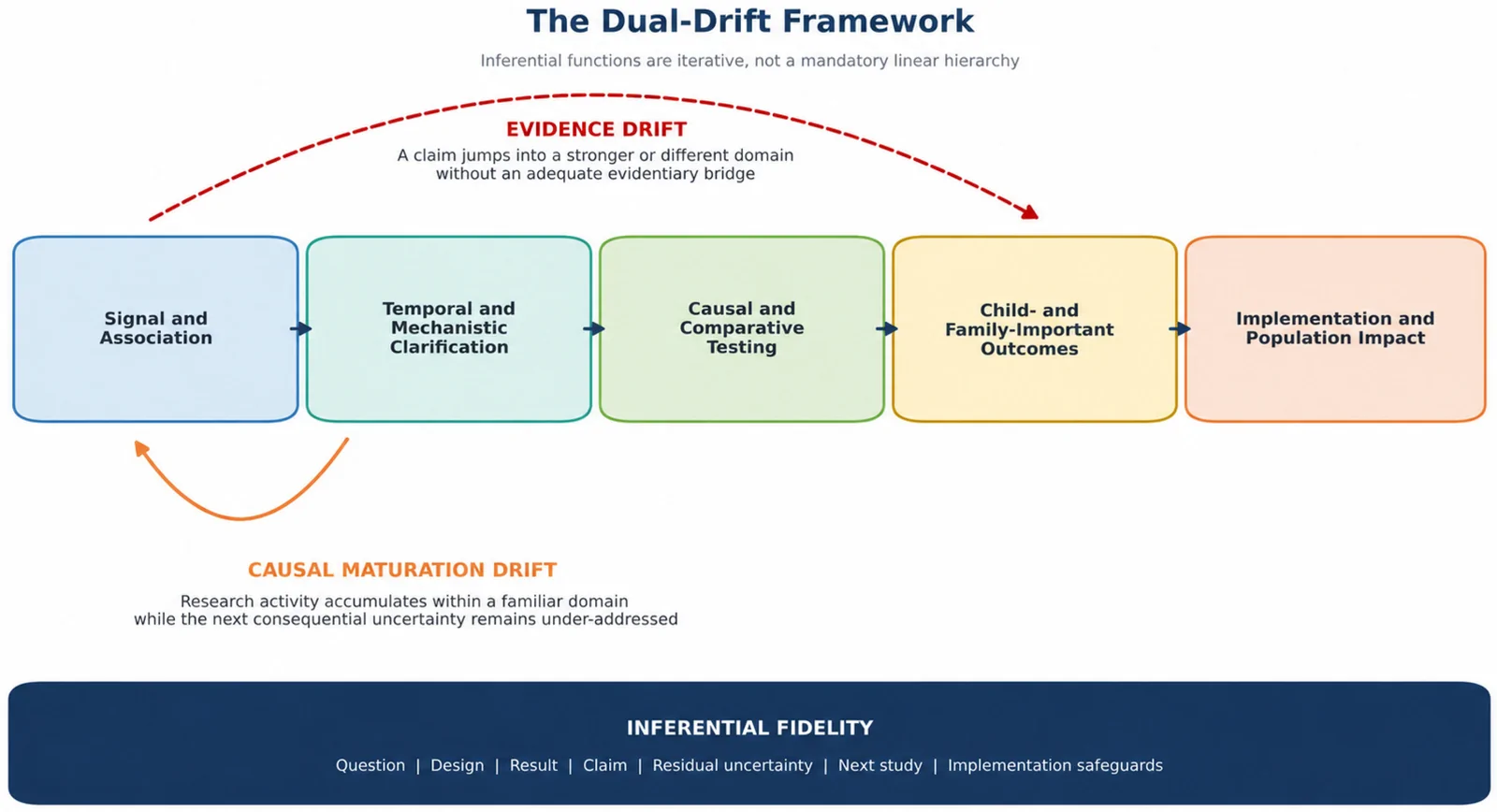
**The Dual-Drift Framework for Pediatric Health Research.** Solid arrows indicate guarded progression toward decision-relevant evidence. The dashed leap indicates Evidence Drift; the looping arrow indicates Causal Maturation Drift. Inferential Fidelity governs both claim calibration and selection of the next consequential research step.

The Dual-Drift Model therefore distinguishes two axes (Figure 1). A field may have low Evidence Drift but high CMD: authors describe associations cautiously, yet the literature remains trapped in repetitive descriptive work. It may have high Evidence Drift but low CMD: a rapidly maturing evidence base is nevertheless communicated with overconfident conclusions. The greatest concern arises when both are high—immature evidence is repeatedly generated and simultaneously translated beyond its limits.

**Figure 2:**
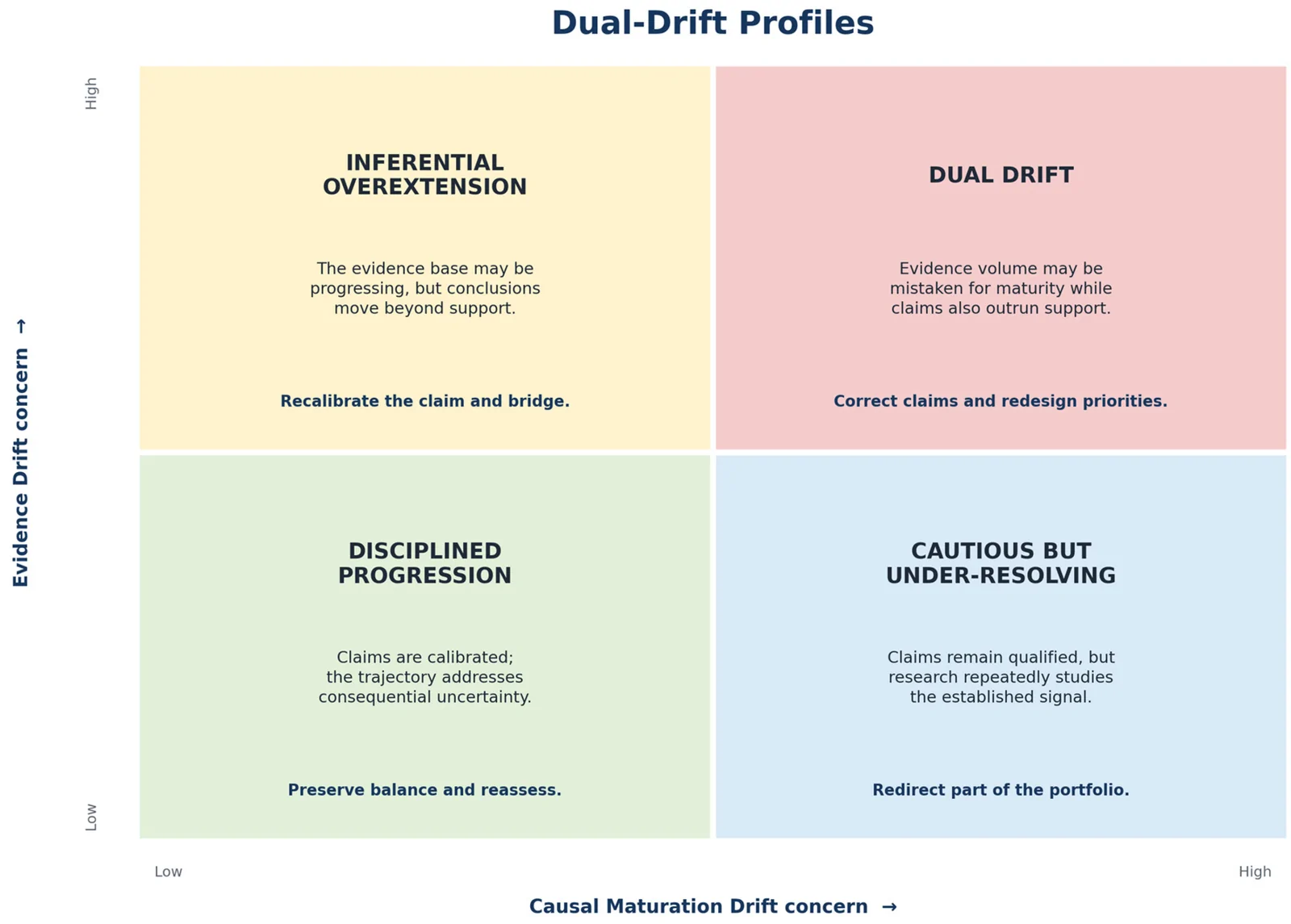
**Dual-Drift profiles in pediatric evidence.** Evidence Drift and Causal Maturation Drift should be evaluated separately because each profile requires a different response. A two-by-two matrix crosses Evidence Drift concerns with Causal Maturation Drift concerns. Quadrants show disciplined progression, inferential overextension, cautious but under-resolving research, and dual-drift.

The four possible combinations of Evidence Drift and Causal Maturation Drift risk, together with their interpretations and corresponding corrective responses, are mapped in Figure 2. This matrix preserves the two drifts as independent dimensions requiring distinct responses: disciplined progression, inferential overextension, cautious but under-resolving research, and dual-drift.

### Distinguishing Claim-Level (EDAS) and Trajectory-Level (CMDAS) Appraisals

Because Evidence Drift and Causal Maturation Drift operate at different units of analysis, a single research document may contain an overextended claim (EDAS concern) while also belonging to a research trajectory trapped in lateral accumulation (CMDAS concern). To prevent double-counting and clarify coding, the corresponding claim-level and trajectory-level evaluation domains are compared in Table 2.

Furthermore, determining the ‘principal residual uncertainty’ for CMDAS depends on the decision context. Clinicians may prioritize comparative effectiveness and long-term outcomes; basic scientists may prioritize biological mechanisms; families may prioritize functional burden and quality of life; and health system planners may prioritize cost, safety, and equity. Before scoring CMDAS, assessors must explicitly declare the target stakeholder decision context, as different decision contexts legitimize different primary uncertainties. When multiple stakeholder groups are relevant, assessors should either prespecify a single primary decision perspective to anchor the appraisal or report parallel CMDAS profiles for each group, rather than imposing a single priority.

## 6. Applications in Pediatric Health Research

The following case illustrations demonstrate how the Dual-Drift Framework and its preliminary appraisal domains can be applied across pediatric etiological, clinical, technological, and implementation research. These examples are intended solely as purposive, pedagogical illustrations to clarify the framework’s conceptual boundaries. They do not constitute formal, systematic literature trajectory mappings or diagnostic evaluations of individual research groups, institutions, or investigators.

### 6.1. Association-Focused ECC and Microbiome Research: A General Issue Illustrated Through the Manitoba Sequence

Cross-sectional microbiome studies can identify ecological differences between children with and without early childhood caries (ECC) and generate biologically plausible hypotheses about microbial dysbiosis. Across the international literature, multiple independent research groups have reported associations between salivary or plaque microbiota, feeding practices, nutritional characteristics, host factors, and ECC status [18,19,20,21,22,23,24,25,26,27]. These studies provide valuable descriptive evidence and an important foundation for hypothesis generation. This sequence is presented solely as an exemplar of a broader structural phenomenon in the epidemiological literature, not as an evaluation of specific investigators or institutional output.

The general inferential concern arises when microbial profiles measured after disease onset are interpreted as indicating disease initiation or identifying preventive targets before temporality and modifiability have been established. A post-disease microbial profile may represent a cause, a consequence, a mediator, a correlate of frequent sugar exposure, or an ecological adaptation to the established lesion environment. Evidence Drift may therefore occur when an association is translated into causal or preventive language without the necessary temporal, mechanistic, or intervention evidence.

At the trajectory level, a hypothesis of Causal Maturation Drift may arise when repeated cross-sectional profiling continues to accumulate while pre-lesion longitudinal research, functional investigation, perturbation studies, and intervention testing to determine whether modifying the microbial ecology changes lesion development remain comparatively limited. This pattern is not presumed merely because multiple association-based studies exist. Formal CMD assessment would require systematic mapping of the complete research trajectory, including its time window, design distribution, independent replication, constraints, and principal unresolved uncertainty.

To make this broader methodological concern concrete, the Manitoba sequence is retained as a bounded, purposively selected local illustration. It was selected because of the author’s professional familiarity with the regional literature, gained from previously working in the same academic department. This proximity is disclosed as a potential source of selection bias and does not confer privileged evidence. All observations are limited to publicly available study designs, reported findings, and visible evidentiary bridges; no inference is made about investigator motives or research quality.

The selected Manitoba publications in Table 3 trace an association-focused line of ECC research, spanning conventional determinants and risk factors as well as nutritional, genetic, and microbiome correlates.

Read together, these studies demonstrate the legitimate descriptive value of association-focused ECC research while also making the remaining inferential boundary visible. Feeding practices, nutritional characteristics, genetic factors, and microbial taxa may be associated with ECC, but the designs in this selected sequence do not, by themselves, determine whether the observed microbial patterns preceded disease, contributed causally to its development, resulted from the established lesion environment, or reflected shared influences such as frequent sugar exposure.

Accordingly, no formal CMDAS score or trajectory-level classification is assigned to the Manitoba program. Instead, the sequence serves as a bounded illustration of the question CMDAS is designed to make explicit: after an association has been repeatedly characterized, has the research trajectory progressed toward the temporal, mechanistic, causal, and intervention evidence needed to resolve this gap? The appropriate response is to preserve the value of the descriptive evidence, retain claims within the domains supported by the designs, and clarify which subsequent studies would most effectively test temporality and modifiability.

### 6.2. Vitamin D and Childhood Caries as an Example of Pediatric Evidence Maturation Through Constraint

Observational associations between vitamin D status and childhood caries have since been re-examined using Mendelian randomization analyses [28,29]. A direct recommendation that supplementation prevents ECC would constitute Evidence Drift unless supported by causal and intervention evidence. A 2-year early-childhood vitamin D_3_ dose-comparison trial also found no difference in later oral health outcomes between the intervention groups [30]. Taken together, these studies constrain, rather than confirm, a broad stand-alone preventive claim [28,29,30].

This example shows why maturation should not be equated with confirmation. The trajectory matured as the claim became more constrained and clinically precise. Continued work may still be justified for severely deficient or biologically distinct subgroups, but the next study should be chosen to address a specific residual uncertainty—not merely because another observational dataset is available. The example is particularly relevant to pediatric research because developmental timing, baseline deficiency, dose, growth, diet, and socioeconomic context may modify the interpretation of an apparently consistent observational association.

### 6.3. Silver Diamine Fluoride in Children: Disease Control, Endpoint Inflation, and Downstream Maturation

Silver diamine fluoride (SDF) has evidence supporting the arrest of cavitated caries lesions, though certainty and applicability vary across comparisons and outcomes [31,32]. Evidence Drift occurs when lesion arrest is extended to claims of etiological prevention, restoration of form and function, completion of care, or proof that a simplified delivery pathway is equitable. These claims require additional diagnostic, restorative, longitudinal, and implementation evidence [33].

In pediatric care, the inferential boundary is especially important because lesion arrest may be only one component of a longer pathway that includes symptoms, function, esthetics, child cooperation, caregiver acceptance, pulpal status, definitive treatment, and continuity of care.

A downstream form of CMD may arise if the literature continues to refine short-term arrest percentages while longer-term recurrence, symptoms, pulpal outcomes, comparative effectiveness, definitive-care completion, caregiver-centered outcomes, and equity receive less attention. This is not associative drift; it is endpoint confinement within the disease-control domain. The correction is not to abandon SDF research but to mature the portfolio toward the outcomes needed to define its proper place in comprehensive care.

### 6.4. Pediatric Biomarker and Omics Pipelines

Pediatric biomarker and omics research offers a clear example of child health innovation beyond dentistry. The broader biomarker literature documents substantial translational loss at each stage, from initial discovery through validation, clinical translation, and real-world implementation [34,35].

In pediatric heart failure and congenital heart disease, research highlights the critical need for age-appropriate reference values, rigorous external validation, and demonstrated clinical utility before candidate biomarkers are incorporated into routine care [36].

Within this domain, Evidence Drift occurs when statistical associations, discrimination, or mechanistic plausibility are presented directly as proof of diagnostic or therapeutic benefit across pediatric age groups. Causal Maturation Drift occurs when discovery and internal validation studies continue to accumulate while developmental validity, external validation, cost-effectiveness, and child- and family-important outcomes remain underdeveloped [34,35,36].

### 6.5. Artificial Intelligence Prediction Studies in Pediatric Care

Artificial intelligence (AI) systems for pediatric diagnosis, prognosis, or risk prediction often show high accuracy on retrospective datasets. However, retrospective performance alone does not establish clinical benefit or safety in real-world pediatric health workflows [37,38,39].

Pediatric AI evaluations have distinct methodological requirements. Assessors must consider age distribution, developmental stage, shifting disease prevalence, site-specific case mix, caregiver-mediated decisions, safety, and equity. Most importantly, prospective studies must assess whether AI integration improves outcomes that matter to children and families.

In pediatric clinical AI, Evidence Drift occurs when retrospective model discrimination is directly translated into claims of improved pediatric care. Causal Maturation Drift occurs when successive model architectures and internal validations accumulate while prospective workflow evaluation, age-related validity, safety monitoring, and long-term child health outcomes remain deferred [37,38,39]. Table 4 integrates the five illustrative trajectories by summarizing their valid contributions, possible forms of Evidence Drift and Causal Maturation Drift, and inferentially faithful corrective directions.

## 7. Preliminary Dual-Drift Appraisal Instruments

### 7.1. Purpose and Scoring Principles

The EDAS and CMDAS are proposed as preliminary structured appraisal profiles rather than validated diagnostic or psychometric scales. Their domains are conceived as formative: each captures a distinct way in which inferential alignment may fail, and the items are not assumed to be interchangeable manifestations of a single latent trait. Consequently, items need not correlate, internal consistency is not a prerequisite for usefulness, and a single critical item may remain consequential even when the remainder of the profile is favorable. The names EDAS and CMDAS are retained as provisional labels, but the instruments should not yet be interpreted as conventional psychometric scales.

The domains are specified a priori as formative components of inferential appraisal, not as reflective indicators of a single latent construct. Validation should therefore prioritize content coverage, scoring reproducibility, hypothesis-based construct testing, decision consequences, and practical utility. Internal consistency and factor structure are not primary validation requirements unless future evidence supports reflective subdimensions within one or both instruments.

For exploratory coding, each applicable item may be rated as 0 (no concern), 1 (partial, ambiguous, or emerging concern), or 2 (clear concern). Genuinely non-applicable items may be omitted with explicit justification. The primary output is the item-level profile accompanied by the unit of analysis, evidence used, rationale for each rating, omitted items, and relevant scientific, ethical, developmental, or feasibility constraints.

A standardized descriptive index may be calculated as 100 × observed item sum ÷ maximum possible sum for applicable items. This index should remain secondary to the item-level profile and must not be interpreted as an interval-level measure, diagnostic classification, or validated threshold. Two different item combinations may produce the same index while representing materially different inferential problems.

EDAS and CMDAS should not be combined into a single score because they evaluate different units. EDAS evaluates a claim or recommendation, whereas CMDAS evaluates a defined and mapped trajectory over time. When a claim can be situated within an eligible trajectory, the two descriptive indices may be displayed as a two-axis Inferential Fidelity Profile, provided that the item-level findings and limitations remain visible.

Item scores are not assumed to be independent. A single underlying inferential problem may appropriately affect several items—for example, expanding a narrow endpoint may raise concerns about domain congruence, outcome scope, uncertainty retention, and implementation proportionality. Assessors should explicitly link such ratings to the same underlying reasoning in their narrative justifications and should not interpret the total as the number of distinct failures. This dependence further supports treating the item-level profile, rather than the standardized index, as the primary output.

### 7.2. Evidence Drift Assessment Scale (EDAS)

The eight preliminary EDAS items, appraisal questions, and scoring anchors are presented in Table 5.

**EDAS interpretation.** The item-level profile is the primary output; the standardized descriptive index is secondary. A descriptive total may be reported when at least six items are applicable, but this minimum is a provisional reporting convention rather than an empirically established adequacy threshold. Because a single inferential overextension may activate several related items, the total should not be interpreted as the number of independent errors. A score of 2 on a single consequential item—such as unsupported causal attribution or unsupported replacement of comprehensive care—should remain visible even when the overall index is modest.

**Pediatric application note.** When applying EDAS to child-health evidence, assessors should explicitly consider adult-to-child extrapolation, inappropriate pooling across developmental stages, reliance on caregiver proxy measures when child-reported outcomes are feasible, and substitution of short-term or surrogate outcomes for durable developmental or functional benefit.

### 7.3. Causal Maturation Drift Assessment Scale (CMDAS)

CMDAS begins with an eligibility gate. The assessor must define the research trajectory and time window, justify that the original signal is sufficiently characterized for the intended decision, and identify the main evidentiary gap. If these conditions cannot be met, the trajectory should be mapped descriptively rather than labeled CMD. Cumulative review methods can help determine when additional same-design studies are likely to yield diminishing information value [40,41]. The eight preliminary CMDAS items, trajectory-level appraisal questions, and scoring anchors are presented in Table 6.

**CMDAS interpretation.** Use the standardized score and item profile only after the eligibility gate is met. At least six scored items are recommended. Ethical constraints, low prevalence, the need for external replication, emerging measurement validity, and the lack of feasible interventions should be documented before interpreting a high score as avoidable drift.

**Pediatric application note.** In pediatric trajectories, legitimate explanations for slower maturation may include age-specific rarity, recruitment and consent challenges, long developmental latency, limited feasibility of experimentation, and the need to establish age-appropriate measurement validity. These constraints should be documented before interpreting a high CMDAS profile as avoidable drift.

### 7.4. Operational Guidance for Threshold Judgments

Greater procedural clarity is needed to apply the CMDAS eligibility gate and to distinguish an emerging concern from a persistent concern. The numerical values below—including study counts, publication proportions, and time windows—are provisional heuristic proposals intended solely to make the assumptions explicit and to facilitate future empirical testing. They are not validated thresholds, consensus standards, or formal decision rules. They should not be used to classify research fields, evaluate investigators, reallocate funding, or support high-stakes decisions. Their interpretation is expected to vary with the condition, population, research maturity, feasibility constraints, expected effect size, and consequences of error.

**A. Judgment 1: Is the signal “sufficiently characterized” for the intended decision? (Eligibility Gate)**

A signal is sufficiently characterized when additional studies using the same design, exposure definition, outcome, and population are unlikely to materially alter the direction or approximate magnitude of the effect for the decision at hand. Assessors should consider:

- **Cumulative meta-analysis:** If the pooled estimate’s 95% confidence interval is narrow enough to exclude clinically or practically meaningful effects (or to establish a stable association), the signal meets the characterization. For example, in the vitamin D–caries trajectory, Mendelian randomization and trials produced pooled estimates that, although not uniformly null, narrowed the plausible effect size, making broad population supplementation claims no longer tenable.
- **Consistency across replications:** If at least three high-quality, independent studies (using similar designs) show consistent direction and magnitude, and additional replications are unlikely to alter the qualitative interpretation, the signal may be deemed characterized.
- **Expert or guideline consensus:** Formal consensus statements, living systematic reviews, or guideline panels that explicitly state that the evidence is sufficient to inform a specific decision (e.g., “no further association studies are needed before moving to intervention testing”) can serve as a valid basis.

**Example (passed gate):** A field has five cross-sectional studies and three prospective cohort studies, all consistently showing that exposure X is associated with outcome Y (OR ~1.5, 95% CI 1.3–1.7). The principal unresolved uncertainty is whether modifying X changes Y. The signal is characterized; the gate is passed, and CMDAS may be applied.

**Example (failed gate):** A novel biomarker has only two small, heterogeneous case–control studies with conflicting results. The signal remains uncharacterized. CMDAS should not be applied; the trajectory is still in early discovery. The assessor should map the evidence descriptively without assigning a drift score.

**B. Judgment 2: When does “emerging concentration” (score 1) become “persistent concentration” (score 2)?**

The distinction hinges on time, proportion, and opportunity.

- **Proportion rule:** Over the most recent 3–5 years, if >60% of publications addressing the core question use the same design, exposure, and outcome without adding a new inferential bridge, score 2 (persistent concentration). If 30–60%, score 1 (emerging); if <30%, score 0 (balanced).
- **Time rule:** If the same major limitation (e.g., cross-sectional design, lack of long-term follow-up) has been explicitly acknowledged in the discussion sections of at least three consecutive review articles or guideline updates spanning more than three years, and no corresponding increase in studies addressing that limitation has occurred, score 2.
- **Opportunity rule:** If feasible methods to address residual uncertainty exist—and have been published in related fields—but are absent from the target trajectory despite repeated calls for them, score 2. If methods are not yet feasible, ethically constrained, or require substantial infrastructure, score 1 or 0, with documented justification.

To further clarify its developmental status, a worked example applying these heuristic thresholds is provided in Supplementary Material S2. This illustrates the appraisal process and the need for validated operational precision during validation.

**C. Handling ambiguous cases**

When thresholds are not clearly met, assessors should:

1. Score conservatively (i.e., 0 or 1) and document the ambiguity in a narrative annex.
2. Seek a second independent scoring, especially for high-stakes applications (e.g., funding portfolio reallocation).
3. Use the item-level profile rather than the total score as the primary appraisal output. A provisional score of 2 on a potentially consequential item—for example, a persistent absence of temporal evidence—should prompt closer examination and narrative justification rather than an automatic classification or corrective action.

### 7.5. Worked Use of the Scales

The scales are intended to separate claim appraisal from trajectory appraisal. A cross-sectional ECC microbiome paper concluding that a microbial signature causes disease would raise EDAS concerns about domain incongruence, causal escalation, uncertainty attenuation, and missing bridging evidence. A ten-year microbiome portfolio dominated by post-disease cross-sectional profiling, with the limitation of temporality repeatedly acknowledged, could raise CMDAS concerns about same-domain accumulation, recurrent limitations, a missing temporal bridge, and limited triangulation. Such a conclusion would remain provisional unless supported by systematic trajectory mapping.

By contrast, the vitamin D–caries trajectory illustrates how Mendelian randomization and intervention evidence can reduce CMDAS concerns, even when those studies weaken the original claim. An SDF policy that equates program reach with complete and equitable care could score highly on EDAS implementation overreach, while the associated research portfolio might show CMDAS endpoint confinement if long-term pathway outcomes remain insufficiently developed.

## 8. Uses in Pediatric Research, Review, Funding, and Policy

For **pediatric investigators**, the Dual-Drift Framework adds a portfolio question to ordinary study design: what relevant gap will this study materially reduce? A technically rigorous study may still have low marginal value if it merely reproduces the established signal, while a more consequential bridge remains untouched. This does not imply that every project must be a trial or an implementation study. It requires an explicit explanation of why the selected design is the appropriate next step.

For **reviewers and editors of pediatric research**, EDAS provides a structured way to assess whether titles, abstracts, conclusions, and recommendations preserve developmental, causal, outcome, and implementation boundaries. Systematic reviewers can go further by mapping the distribution of designs and outcomes over time and noting whether the same unresolved limitation recurs.

For **child-health funders and research networks**, CMDAS may support portfolio mapping by identifying imbalances among descriptive discovery, longitudinal research, causal testing, comparative effectiveness, long-term outcomes, and implementation. Such use should guide priorities rather than punish individual researchers.

**Figure 3:**
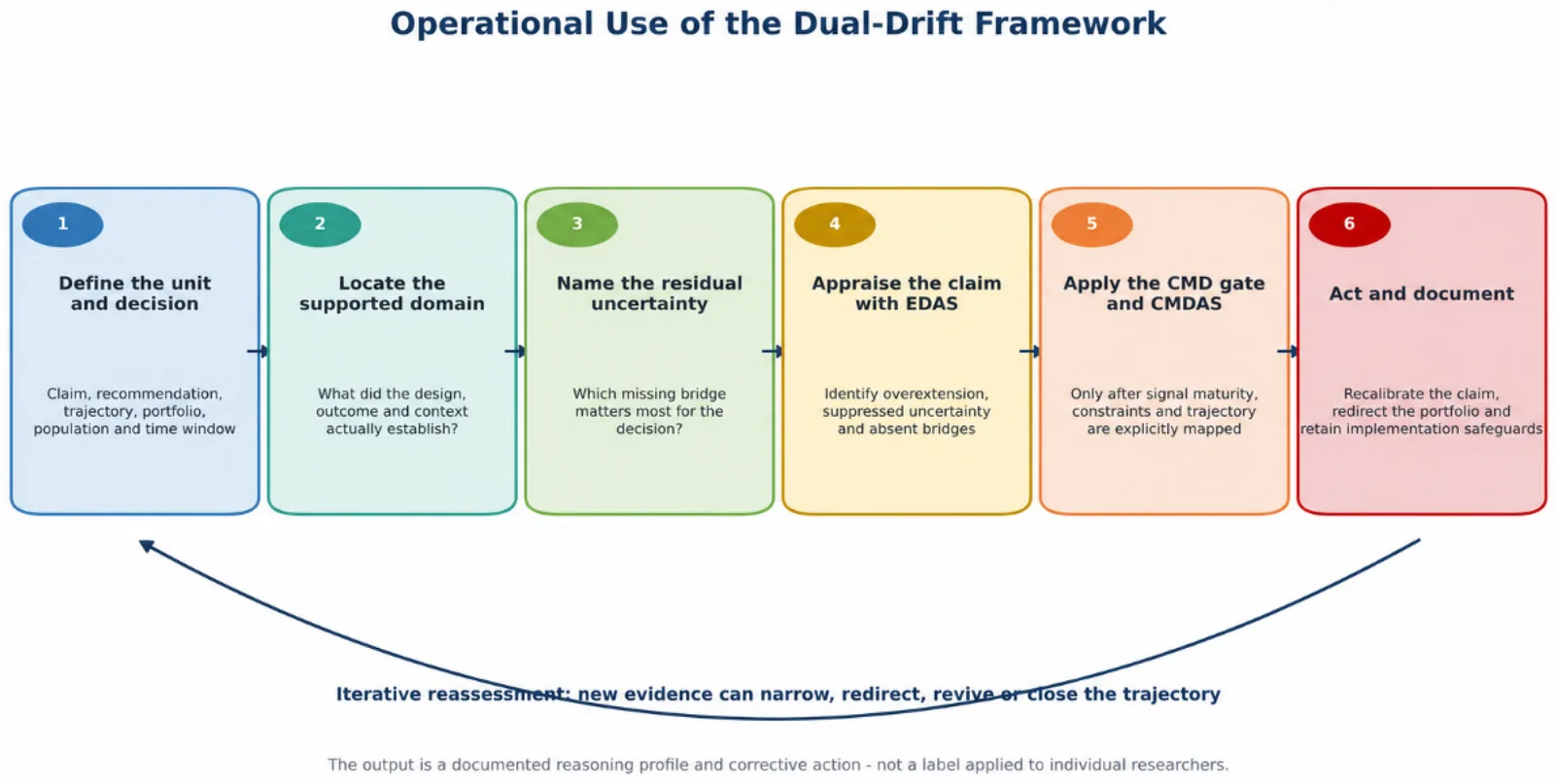
**Operational use of the Dual-Drift Framework in pediatric research.** Appraisal begins by defining the decision and supported domain, then identifies the residual uncertainty, applies the appropriate claim-level or trajectory-level tool, and documents corrective action. Six-step workflow: define the unit and decision, locate the supported domain, name residual uncertainty, apply EDAS, apply the CMD eligibility gate and CMDAS, then recalibrate claims or redirect portfolios with iterative reassessment.

For **pediatric guideline panels and policymakers**, the framework separates evidence of efficacy from evidence that an intervention can be delivered safely, equitably, and developmentally appropriately across child populations and care settings. Implementation frameworks such as RE-AIM and CFIR already emphasize reach, effectiveness, adoption, implementation, context, and maintenance [42,43]. The Dual-Drift contribution is earlier and complementary: before asking how an intervention should be scaled, decision-makers should verify that the clinical or policy claim being implemented is supported and that unresolved evidence has not been mistaken for maturity simply because the literature is large (Figure 3).

In pediatric health, the framework may also help prevent adult evidence, surrogate outcomes, technical performance, or program reach from being substituted for evidence of developmentally appropriate benefit. Its use should foreground child-reported outcomes where feasible, caregiver perspectives where necessary, long-term safety, continuity of care, and equity for socially or medically vulnerable children.

To demonstrate the operational use of the preliminary instruments without overstating their validation, Supplementary Material S1 applies EDAS to three synthetic SDF claim formulations and uses the pediatric SDF literature to illustrate the CMDAS eligibility and mapping process. The supplement provides item-level EDAS scores, demonstrates gradations among evidence-calibrated, boundary-blurring, and clearly overextended claims, and specifies the evidence required before a formal trajectory-level CMDAS score can be assigned. Because the SDF research trajectory has not been systematically mapped in this concept paper, no formal CMDAS total or Dual-Drift classification is reported. The worked example is pedagogical and does not constitute a systematic assessment of the SDF literature or its investigators.

The following hypothetical but practice-oriented examples illustrate how the framework could be applied after appropriate validation:

**Example 1. Research funders and grant panels.** A pediatric funding agency reviewing a portfolio of childhood asthma biomarker research finds that discovery and retrospective validation studies dominate, while prospective clinical-utility studies and age-stratified external validation remain limited. Rather than treating publication volume as evidence of maturity, the panel uses the provisional CMDAS domains to identify the unresolved bridge and issues a funding call for prospective validation, clinical-utility evaluation, and implementation-safety research. At this developmental stage, this would constitute a structured portfolio discussion rather than a validated CMD classification.

**Example 2. Guideline panels and journal editors**. A pediatric dental guideline panel evaluates a proposed recommendation that presents silver diamine fluoride as a replacement for long-term comprehensive care. The panel recognizes that evidence of lesion arrest supports a lesion-level disease-control claim but, by itself, does not establish restoration of form and function, completion of care, long-term safety, or equitable implementation. Using the EDAS domains, the panel recalibrates the recommendation to preserve the demonstrated endpoint and explicitly identifies the comparative, longitudinal, patient-centered, and implementation evidence required before broader claims can be made. This example illustrates claim calibration rather than a validated EDAS classification.

## 9. Validation Roadmap

The proposed scales require empirical development before they are used for formal judgments. Initial validation should be conducted within pediatric health research, the primary intended context of use. Cross-disciplinary transferability should be examined only after the constructs demonstrate acceptable content validity, reliability, and practical utility in child-health samples.

A phased empirical development program is required before these provisional appraisal domains can be considered validated meta-research instruments. The roadmap is informed, where applicable, by established guidance for developing and evaluating measurement instruments, including general scale-development recommendations and the COSMIN (COnsensus-based Standards for the selection of health Measurement INstruments) framework [44,45]. Because EDAS and CMDAS are meta-research appraisal tools rather than patient-reported health-status measures, the relevance of individual COSMIN standards should be evaluated rather than assumed.

**Phase 1: Content Validity and Domain Refinement.** Expert Delphi panels comprising pediatric investigators, biostatisticians, epidemiologists, methodologists, journal editors, guideline developers, and patient or caregiver representatives will assess item relevance, comprehensiveness, comprehensibility, and usability. Content-validity procedures will be informed by COSMIN guidance and adapted to the meta-research purpose of EDAS and CMDAS [45]. Cognitive interviews with intended users will examine how each item is interpreted, response processes, and sources of ambiguity before formal reliability testing.

**Phase 2: Feasibility, Inter-Rater Reliability, and Multi-Assessor Testing.** Pilot testing will involve independent multi-assessor scoring across diverse pediatric studies. Studies will report item-level weighted kappa or another agreement coefficient appropriate for ordinal ratings, agreement on the CMDAS eligibility-gate decision, and, if the secondary standardized index is examined, an appropriate intraclass correlation coefficient. Completion time, sources of disagreement, adjudication reasoning, and performance across assessor groups and pediatric domains will also be reported.

**Phase 3: Hypothesis-Based Construct Evaluation and Trajectory Mapping**. Empirical testing will use prespecified hypotheses to determine whether EDAS profiles distinguish claims independently judged to remain within, blur, or cross inferential domains, and whether CMDAS profiles distinguish mapped trajectories that do or do not reduce a declared residual uncertainty. Analyses will assess known-groups discrimination, convergence with relevant assessments of spin, indirectness, and research-priority alignment, discrimination between EDAS and CMDAS, sensitivity to stakeholder decision context, and robustness across pediatric domains [46,47]. Factor analysis will not be treated as a required validation criterion because the items are specified as formative; it will be considered only if later evidence supports reflective subdimensions.

**Phase 4: Decision Consequences and Practical Utility.** Prospective evaluations will assess completion time, usability, effects on reviewer and funding judgments, reproducibility of corrective actions, and unintended consequences. Bibliometric trajectory-mapping studies will assess whether CMDAS profiles align with observable changes in study designs, outcomes, and evidentiary bridges over time.

## 10. Limitations and Safeguards Against Misuse

The framework is conceptual, and the scales are unvalidated. The threshold for a signal to be “sufficiently characterized” will vary by decision, population, expected effect size, measurement quality, and consequences of error. Some trajectories mature slowly for legitimate reasons, including ethical limits on experimentation, rare outcomes, long latency, inadequate measurement, and the absence of feasible interventions.

Pediatric research trajectories may mature slowly for legitimate reasons, including age-specific rarity, recruitment and consent challenges, developmental changes in exposure and outcome measurement, long latency between childhood exposure and later consequence, and restricted feasibility of experimentation. The framework should therefore distinguish avoidable under-resolution from scientifically or ethically necessary caution.

The framework should not be used retrospectively to imply negligence or motive. The examples are selective and do not constitute systematic reviews of the fields represented. Formal CMD assessment requires explicit trajectory mapping and should not be inferred merely from the perception that “many similar papers” exist. Moreover, research maturation is iterative: implementation may reveal new mechanisms, null trials may revive measurement questions, and subgroup findings may justify renewed association work. The model therefore rejects a rigid ladder while demanding clarity about the next consequential uncertainty.

Although the constructs are conceptually portable, the present paper develops and illustrates them primarily within pediatric health research. Their applicability to adult medicine, other biomedical fields, or non-health disciplines should be treated as an empirical question rather than assumed.

Finally, scale scores should not be used to rank individual scientists, journals, or disciplines until validated. The appropriate target is the alignment of claims and portfolios, not blame. Item profiles may ultimately be more informative than total scores because a single severe inferential leap or a single persistently neglected bridge can be consequential even when other dimensions are strong.

## 11. Conclusions

Scientific progress in pediatric health can fail in two opposing ways: claims may exceed the evidence supporting them, or research activity may expand without resolving the uncertainty most relevant to children, families, and clinical decisions. Evidence Drift captures the first failure at the level of a claim or recommendation. Causal Maturation Drift captures the second at the level of a defined research trajectory. Inferential Fidelity unifies these constructs by requiring alignment among the question, design, results, claim, residual uncertainty, subsequent research, and safeguards required for implementation.

The Dual-Drift Framework and its preliminary appraisal domains offer a testable conceptual starting point for investigators, reviewers, editors, funders, guideline panels, and policymakers. The framework is not yet a validated measurement system: its items, scoring structure, numerical heuristics, reliability, validity, and interpretive thresholds require empirical evaluation. Its immediate contribution is therefore conceptual and organizational—making claim-level overextension and trajectory-level under-resolution visible as related yet analytically distinct problems.

Scientific progress therefore requires both calibrated claims and research portfolios focused on consequential uncertainty. Although developed for pediatric health research, the framework’s transferability to adult medicine, other biomedical fields, and non-health disciplines remains an empirical question that should be tested rather than assumed. Testing cross-disciplinary transferability in adult clinical medicine, oncology, or the social sciences is left for future work.

## Figures and Tables

**Table 2:** Paired Comparison of Claim-Level (EDAS) and Trajectory-Level (CMDAS) Items.

Evaluation Focus	Claim-Level Item (EDAS)	Trajectory-Level Item (CMDAS)	Practical Coding Distinction
Causal/Temporal Bridge	*Item 8: Bridging Evidence Transparency* (Does a single report identify the evidence required before advancing a stronger claim?)	*Item 4: Missing Temporal/Causal Bridge* (Are designs capable of testing temporality, confounding, or counterfactuals absent across the literature?)	EDAS checks if *one document* acknowledges its inferential limit; CMDAS checks if *the field over time* fails to execute bridging studies.
Endpoint/Outcome Scope	*Item 4: Outcome-Scope Inflation* (Does a single report extrapolate a short-term/surrogate outcome into disease resolution or comprehensive care?)	*Item 6: Endpoint Confinement* (Does the broader literature remain persistently focused on surrogate/technical endpoints over years?)	EDAS flags *over-claiming* based on a narrow endpoint; CMDAS flags *failure of the field* to develop patient-important outcomes.
Implementation	*Item 7: Implementation/Policy Proportionality* (Does a report equate short-term efficacy or uptake with real-world safety, equity, or replacement of care?)	*Item 7: Translation/Implementation Deferral* (Are comparative effectiveness, safety, equity, and delivery repeatedly postponed over time?)	EDAS catches *premature claims* of real-world implementation; CMDAS catches *persistent delay* in conducting real-world delivery research.

**Table 3:** Selected Manitoba Publications Used as a Bounded Illustration of an Association-Focused ECC Research Sequence.

Timeframe	Study	Design	Primary Association or Domain Examined
2005	Schroth & Moffatt [21]	Cross-sectional pilot	Determinants of ECC in rural Manitoba
2007	Schroth & Cheba [22]	Cross-sectional	Prevalence and risk factors in a community dental clinic
2025	Lee et al. [23]	Case–control	Nutritional status and *Veillonella* with caries
2025	Khan et al. [24]	Case–control	Taste genetics and dental plaque microbiome
2026	Szeto et al. [20]	Cross-sectional	Feeding practices, *Rothia*, and ECC

Note: Publications were selected because they arose from the same regional research environment, used association-focused designs to examine ECC, and spanned the development from conventional risk-factor studies to recent microbial and genetic analyses. The sequence is purposive and non-exhaustive; it neither represents the complete Manitoba research program nor establishes Evidence Drift or Causal Maturation Drift. Formal CMDAS appraisal would require a reproducible search, systematic mapping of the complete eligible trajectory, explicit satisfaction of the eligibility gate, and independent assessment.

**Table 4:** Applications of the Dual-Drift Framework in Pediatric Health Research.

Trajectory	Valid Contribution	Evidence Drift	Causal Maturation Drift	Inferentially Faithful Correction
ECC microbiome	Describes microbial ecology and generates hypotheses concerning possible disease-related pathways	A post-disease microbial profile is presented as proof of disease initiation or as an established preventive target	Could be suspected if repeated profiling substantially outpaces pre-lesion longitudinal, functional, perturbation, and intervention research	Preserve the association-level boundary; systematically map the trajectory before making a CMD judgment; prioritize temporality and modifiability
Vitamin D and caries	Identifies an observational signal and plausible pathways	Association is translated directly into supplementation advice	Would occur if association studies continued without causal tests	Use triangulation and trials; allow the hypothesis to be narrowed or weakened
SDF	Estimates lesion-arrest effects within disease control	Arrest is presented as prevention, restoration, or completion of care	Short-term arrest remains dominant while long-term pathway outcomes lag	Preserve the endpoint boundary and mature research toward comparative, patient-centered, and implementation outcomes
Pediatric biomarkers/omics	Discovers candidate markers for childhood disease, prognosis, treatment response, or developmental outcomes	Association or discrimination is presented as clinical utility across pediatric age groups	Discovery proliferates without developmental, external, and clinical-utility validation	Validate across developmental stages and settings; test incremental value and child- and family-important outcomes
Pediatric clinical AI	Develops prediction or decision-support systems for pediatric care	Retrospective performance is presented as improved pediatric care	Model development accumulates while prospective child-health evaluation is delayed	Evaluate live workflow, age-related validity, safety, fairness, decisions, and child outcomes

**Table 5:** Preliminary Evidence Drift Assessment Scale (EDAS).

Item	Appraisal Question	Scoring Anchors
1. Domain congruence	Does the conclusion answer the same inferential question that the study or evidence synthesis actually addressed?	0 = Same domain 1 = Boundary is blurred 2 = Stronger or different domain
2. Causal escalation	Is causal language proportionate to temporality, confounding control, identification assumptions, and the design?	0 = Proportionate 1 = Partly overstated or unclear 2 = Association presented as causation
3. Mechanism or surrogate substitution	Is biological plausibility, a biomarker, or an intermediate outcome used as evidence of clinical or population benefit?	0 = No substitution 1 = Qualification incomplete 2 = Substitution is explicit or functionally implied
4. Outcome-scope inflation	Is a narrow, lesion-level, short-term, or process outcome expanded to disease resolution, comprehensive care, or patient benefit?	0 = Outcome boundary retained 1 = Some inflation 2 = Clear inflation
5. Contextual extrapolation	Are population, setting, exposure range, comparator, follow-up, or delivery conditions generalized beyond the support?	0 = Context retained 1 = Limited unsupported extrapolation 2 = Broad unsupported extrapolation
6. Uncertainty retention	Does the conclusion preserve relevant uncertainty, alternative explanations, heterogeneity, and limitations?	0 = Preserved 1 = Selectively attenuated 2 = Material uncertainty suppressed
7. Implementation/policy proportionality	Are efficacy, uptake, or reach equated with real-world effectiveness, sustainability, safety, equity, or replacement of the standard of care?	0 = No overreach 1 = Partial overreach 2 = Clear overreach
8. Bridging evidence transparency	Does the report identify what additional evidence would be required before advancing the stronger claim?	0 = Bridge explicit 1 = Bridge vague 2 = Bridge absent while a stronger claim is advanced

**Table 6:** Preliminary Causal Maturation Drift Assessment Scale (CMDAS).

Item	Trajectory-Level Appraisal Question	Scoring Anchors
Eligibility gate (not scored)	Are the trajectory, time window, maturity of the initial signal, and the principal unresolved uncertainty explicitly defined?	Proceed only when adequately specified. Otherwise, map the evidence without assigning a drift score.
1. Same-domain accumulation	After signal establishment, do similar designs, exposures, populations, or outcomes continue to dominate?	0 = Balanced progression 1 = Emerging concentration 2 = Persistent concentration
2. Marginal uncertainty reduction	Do later studies add detail or precision but make little progress on the primary unresolved uncertainty?	0 = Material reduction 1 = Mixed contribution 2 = Predominantly marginal
3. Recurrent unresolved limitation	Does the same major limitation recur across publications without being directly addressed?	0 = Limitation addressed 1 = Partial progress 2 = Persistent recurrence
4. Missing temporal/causal bridge	Are designs capable of clarifying temporality, confounding, intervention effects, or counterfactual contrasts delayed or underrepresented?	0 = Appropriate bridge evidence present 1 = Incomplete 2 = Persistently underrepresented
5. Limited triangulation	Does the field rely heavily on methods that share similar biases rather than combining approaches with different bias structures?	0 = Strong triangulation 1 = Limited triangulation 2 = Single-method dependence
6. Endpoint confinement	Does research remain focused on surrogate, short-term, technical, or narrowly defined outcomes despite broader clinical questions?	0 = Broader outcomes developed 1 = Partial confinement 2 = Persistent confinement
7. Translation/implementation deferral	When evidence maturity warrants it, are comparative effectiveness, implementation, safety, sustainability, and equity repeatedly postponed?	0 = Timely progression 1 = Partial deferral 2 = Persistent deferral
8. Portfolio alignment	Do funding and publication patterns remain disproportionately aligned with an established signal or with available technology rather than with the core gap?	0 = Aligned with key uncertainty 1 = Mixed alignment 2 = Clear imbalance

## Data Availability

No new data were created or analyzed in this study. Data sharing is not applicable to this article.

## Supplementary Materials

The following supporting information can be downloaded at https://www.mdpi.com/article/10.3390/children13091161/s1, Supplementary Material S1: Illustrative Worked Application of the Preliminary Dual-Drift Appraisal Instruments to Silver Diamine Fluoride in Pediatric Dentistry. Supplementary Material S2: Application of Provisional Heuristic Thresholds for the Causal Maturation Drift Assessment Scale (CMDAS).

## Abbreviations

**ED**	Evidence Drift
**CMD**	Causal Maturation Drift
**EDAS**	Evidence Drift Assessment Scale
**CMDAS**	Causal Maturation Drift Assessment Scale
**ECC**	Early Childhood Caries
**SDF**	Silver Diamine Fluoride
**AI**	Artificial Intelligence
**RE-AIM**	Reach, Effectiveness, Adoption, Implementation, Maintenance
**CFIR**	Consolidated Framework for Implementation Research

**Glossary of Key Terms**

**Term**	**Definition**
**Associative saturation**	Repeated exposure–outcome studies conducted after the association has already been characterized by sufficient stability for the decision at hand; a manifestation of Causal Maturation Drift.
**Causal escalation**	Describing an association as causation without an adequate identification strategy (e.g., no temporality, no control for confounding, and no explicit assumptions). A form of Evidence Drift.
**Causal Maturation Drift (CMD)**	A persistent trajectory-level pattern in which a field continues to generate, replicate, or refine findings within an established narrow domain, without proportionate progress toward the temporal, mechanistic, causal, comparative, long-term, or implementation evidence needed to resolve the principal decision-relevant uncertainty.
**Contextual extrapolation**	Generalizing findings from one population, setting, exposure range, comparator, or follow-up period to another without empirical or theoretical support. This is a form of Evidence Drift.
**Dual-Drift Framework**	A conceptual model that distinguishes two complementary failures—Evidence Drift (inferential overextension) and Causal Maturation Drift (developmental under-resolution)—unified under the principle of Inferential Fidelity.
**Endpoint confinement**	Persistent focus on a narrow, surrogate, short-term, or technical outcome, while patient-important, comparative, or long-term outcomes remain underdeveloped. A manifestation of Causal Maturation Drift.
**Endpoint inflation**	Expanding a narrow, lesion-level, short-term, or process outcome into a claim of disease resolution, comprehensive benefit, or patient-important outcome. This is a form of Evidence Drift.
**Evidence Drift (ED)**	The movement of a finding toward a stronger or different causal, clinical, implementation, or policy claim without an adequate evidentiary bridge. Assessed at the claim level.
**Evidence-Implementation Fidelity**	The preservation of diagnostic accuracy, monitoring, safety, referral pathways, patient-centered outcomes, and equity when translating evidence into real-world delivery. A dimension of Inferential Fidelity.
**Implementation deferral**	Repeated postponement of studies on delivery, safety, sustainability, equity, or comparative effectiveness after efficacy is established. A manifestation of Causal Maturation Drift.
**Implementation overreach**	Equating efficacy, uptake, or reach with proof of real-world effectiveness, sustainability, safety, equity, or replacement of the standard of care is a form of Evidence Drift.
**Inferential Fidelity**	The preservation of alignment among the question posed, the design used, the result obtained, the claim advanced, the uncertainty that remains, the next research step selected, and the safeguards maintained during implementation.
**Lateral accumulation**	Growth in publications, datasets, or analytical detail that does not proportionally improve the resolution of the central inferential or clinical question. The defining movement of Causal Maturation Drift.
**Mechanistic recursion**	Increasingly detailed laboratory or omics explanations that lack sufficient linkage to temporality, intervention, or clinically modifiable targets. This is a manifestation of Causal Maturation Drift.
**Mechanistic substitution**	Treating biological plausibility or a biomarker as proof that modifying a mechanism improves patient or population outcomes. A form of Evidence Drift.
**Question-Design Fidelity**	The extent to which the chosen study design can validly answer the stated research question. A dimension of Inferential Fidelity.
**Result-Claim Fidelity**	The proportionality between the evidence obtained (including its limitations) and the conclusion or recommendation advanced. A dimension of Inferential Fidelity.
**Scientific maturation (functional definition)**	Reducing consequential uncertainty, clarifying the conditions under which a claim is valid, or demonstrating that a hypothesis should be narrowed, redirected, or abandoned. Maturation is not equated with confirmation.
**Technology-led refinement**	Use of a new platform to generate more granular versions of an established observation without answering the next clinical or inferential question. This is a manifestation of Causal Maturation Drift.
**Triangulation**	The use of multiple approaches with different, preferably unrelated, sources of bias to address the same causal or clinical question strengthens inference when findings converge.
**Uncertainty-Next-Study Fidelity**	The alignment of subsequent research with the most consequential unresolved issue, rather than merely reproducing the most accessible finding. This is a dimension of Inferential Fidelity.
