# Supplementary material for: Evidence Drift and Causal Maturation Drift in Pediatric Health Research: A Dual-Drift Framework and Preliminary Appraisal Instruments for Inferential Fidelity
Source: Children (Basel). 2026 Aug 28;13(9):1161. doi: 10.3390/children13091161 (PMC13605608; doi:10.3390/children13091161)
Supplement: Supplementary file 1 [file children-13-01161-s001.zip › children-4470704-supplementary.pdf]

Supplementary Material S1

Illustrative Worked Application of the Preliminary Dual-Drift Appraisal Instruments to Silver Diamine Fluoride in Pediatric Dentistry

Interpretive rule

This supplement demonstrates the proposed appraisal instruments; it does not validate them. EDAS results are descriptive and uncategorized. Because the SDF literature has not been systematically mapped here, no formal CMDAS total or Dual-Drift classification is assigned.

A. Purpose and Scope

This supplement demonstrates how the proposed Evidence Drift Assessment Scale (EDAS) may be applied to a specific claim and how the proposed Causal Maturation Drift Assessment Scale (CMDAS) may structure the mapping of a research trajectory.

The worked example is pedagogical. It is not a systematic review, bibliometric analysis, or formal diagnosis of the SDF field. Reference numbers correspond to the main manuscript. Because CMDAS evaluates a literature trajectory rather than a single statement, the present supplement uses CMDAS to identify testable trajectory-level concerns and the evidence required to score them formally. It does not present unsupported publication proportions, validated cut points, or categorical labels such as “high” or “low.”

B. Decision Context and Supported Evidence Domain

Table S1. Decision context for the worked example

Element	Specification
Decision question	What does the current evidence permit an author, guideline panel, or policymaker to claim about pediatric SDF?
Supported evidence domain	Professionally applied SDF can increase arrest of some cavitated caries lesions over short-term follow-up, although certainty, comparator, lesion characteristics, application schedule, and applicability vary [31,32].
Claims not established by lesion-arrest evidence alone	Complete management of the child’s caries disease; restoration of form, function, or esthetics; replacement of comprehensive restorative care; long-term comparative effectiveness; completion of definitive care; or equitable population implementation [31–33].
Unit assessed by EDAS	A defined claim or recommendation.
Unit assessed by CMDAS	A systematically defined literature trajectory or research portfolio over time.
Assessment date	August 2026.

C. Evidence Drift Assessment Scale (EDAS): Worked Claim

Claim under assessment:

> *"Silver diamine fluoride is an effective, minimally invasive treatment for childhood caries that can replace conventional restorative care for many children and improve access in underserved communities."*

Source type: Synthetic statement representing a plausible policy or promotional claim; it is not attributed to any individual article, organization, or investigator.

Scoring anchors: 0 = no concern; 1 = partial, ambiguous, or emerging concern; 2 = clear concern. The score is illustrative and reflects one assessor's application of an unvalidated instrument.

Table S2. Illustrative EDAS item profile for the target claim

Item	Appraisal question	Score	Illustrative justification
1. Domain congruence	Does the conclusion remain within the inferential domain supported by the evidence?	2	The supported outcome is lesion arrest. The claim expands this to treatment of childhood caries as a whole, replacement of restorative care, and improved access.
2. Causal escalation	Is causal language proportionate to the design and evidence?	1	Randomized and synthesized evidence can support causal language for a specific lesion-arrest effect in defined comparisons [31,32]. The causal extension to reduced restorative need and improved access is not established.
3. Mechanism or surrogate substitution	Is biological plausibility, a biomarker, or an intermediate outcome used as evidence of broader clinical or population benefit?	1	Lesion arrest is a direct lesion-level outcome, not merely a mechanistic marker; however, the claim functionally uses it as a proxy for completion of comprehensive care.
4. Outcome-scope inflation	Is a narrow or short-term outcome expanded to disease resolution or comprehensive benefit?	2	A lesion-level, mainly short-term outcome is expanded to comprehensive disease management and treatment substitution.
5. Contextual extrapolation	Are population, setting, comparator, follow-up, or delivery conditions generalized beyond support?	1	The claim extends across children, settings, lesion characteristics, delivery models, and underserved populations without specifying the conditions represented in the evidence.
6. Uncertainty retention	Does the claim preserve relevant uncertainty and limitations?	2	The statement does not retain uncertainty about evidence certainty, variable applicability, repeated application, monitoring, long-term outcomes, or downstream care.
7. Implementation/policy proportionality	Is lesion-level efficacy equated with real-world effectiveness, access, equity, or replacement of care?	2	Lesion-arrest efficacy is equated with service access and a change in the care pathway. Those conclusions require separate implementation and equity evidence [33].
8. Bridging-evidence transparency	Does the claim identify the evidence required before stronger conclusions are advanced?	2	The claim does not identify the comparative, longitudinal, pathway, implementation, or equity evidence required to support its broader conclusions.

Descriptive EDAS result

Item profile: 2, 1, 1, 2, 1, 2, 2, 2. Raw total: 13/16; standardized total: 81.25%. The numerical result is not a validated category. The most consequential concerns are domain crossing, outcome-scope inflation, uncertainty attenuation, implementation overreach, and absence of an explicit evidentiary bridge.

D. Contrast Set: Demonstrating Gradation Rather Than Only Extremes

The following synthetic statements are included to show how EDAS may distinguish calibrated, boundary-blurring, and clearly overextended claims. Totals are illustrative one-assessor ratings and should not be interpreted as validated thresholds.

Table S3. Illustrative EDAS profiles across three claim formulations

Claim formulation	Illustrative item profile (Items 1–8)	Illustrative total	Main flags	Interpretive note
Calibrated claim: "SDF can arrest some cavitated caries lesions in children over short-term follow-up, although certainty and applicability vary across comparisons and clinical contexts. It should be used within a diagnosis-led, monitored care pathway and does not by itself establish completion of comprehensive care [31–33]."	0, 0, 0, 0, 0, 0, 0, 0	0/16	None under the proposed anchors	The outcome, context, uncertainty, and care-pathway boundary are retained.
Boundary-blurring claim: "SDF is an effective minimally invasive option for managing cavitated caries lesions and may reduce the immediate need for operative treatment in selected children."	1, 0, 0, 1, 0, 1, 0, 1	4/16	Domain congruence; outcome scope; uncertainty; bridge	The words "managing" and "reduce the immediate need" require clearer conditions, monitoring, and a statement of what remains unresolved.
Overextended target claim used in Table S2	2, 1, 1, 2, 1, 2, 2, 2	13/16	Multiple claim-level boundaries	The claim converts lesion arrest into comprehensive treatment substitution and a system-level access conclusion.

E. Causal Maturation Drift Assessment Scale (CMDAS): Eligibility and Mapping Decision

CMDAS asks whether a defined body of research has continued to concentrate on an established signal while the highest-value causal or decision-relevant uncertainty remains unresolved. A defensible score requires a protocol, a complete or reproducible publication set, explicit coding of designs and outcomes, and a stated basis for judging signal maturity. Those elements are not provided by this concept-paper supplement.

Table S4. CMDAS eligibility and assessment-sufficiency check

Criterion	Status	Justification
Trajectory and time window defined	Partly met	Provisional focus: contemporary pediatric SDF literature represented by a recent systematic review, pediatric guideline, and related policy analysis [31–33]. A comprehensive publication set and reproducible search are not available.
Initial signal sufficiently characterized for the intended decision	Provisionally met	The lesion-arrest signal is sufficiently developed for an illustrative question about short-term disease control [31,32]. It is not sufficient for claims about comprehensive care, long-term comparative effectiveness, or equitable implementation.

Criterion	Status	Justification
Principal unresolved uncertainty identified	Met	Candidate uncertainties include long-term clinical and pathway outcomes, comparative effectiveness, definitive-care completion, child- and family-important outcomes, implementation, safety, sustainability, and equity.
Legitimate ethical and feasibility constraints considered	Partly met	Extended follow-up and real-world implementation studies require resources and may face retention challenges. The magnitude of these constraints must be documented rather than assumed.
Systematic trajectory mapping available	Not met	No registered protocol, comprehensive search, coded denominator, publication-by-publication trajectory map, funding map, or independent ratings are included.
Application decision	Formal score withheld	Use CMDAS here as a structured concern map and methods plan only. Do not assign a total, a categorical label, or a formal diagnosis of Causal Maturation Drift.

F. CMDAS Concern Map and Evidence Required for Formal Scoring

Table S5. Testable CMDAS hypotheses for a future systematic SDF trajectory assessment

CMDAS item	Preliminary concern to test	Evidence required before scoring
1. Same-domain accumulation	Short-term lesion-arrest outcomes may remain a large share of the recent SDF portfolio.	Conduct a systematic search; classify every eligible publication by design, comparator, population, endpoint, and follow-up; report proportions and sensitivity analyses. Do not substitute an unsupported percentage.
2. Marginal uncertainty reduction	Later studies may refine concentration, application frequency, or subgroup estimates without materially reducing downstream uncertainty.	Predefine the principal unresolved uncertainty with clinical, methodological, caregiver, and youth input; then code the incremental contribution of each study.
3. Recurrent unresolved limitation	Short follow-up, limited comparative evidence, and implementation gaps may recur across reviews and guidelines.	Code limitations and research recommendations in consecutive systematic reviews and guideline updates; assess whether corresponding bridge studies subsequently increased.
4. Missing temporal/causal bridge	Long-term comparative and pathway studies may be underrepresented relative to short-term lesion outcomes.	Map follow-up duration, comparators, recurrence, symptoms, pulpal outcomes, adverse events, and completion or transition to definitive care.
5. Limited triangulation	Designs may vary while relying on the same narrow endpoint and related bias structures.	Map study designs by the inferential question they address and by their main bias structures; evaluate whether different methods converge on consequential outcomes.
6. Endpoint confinement	Lesion arrest may receive greater attention than symptoms, function, esthetics, oral-health-related quality of life, caregiver acceptability, pulpal status, and care completion.	Develop an outcome taxonomy and report the frequency and temporal trend of lesion-level, child-reported, family-reported, clinical, and pathway outcomes.
7. Translation/implementation deferral	Real-world delivery, training, fidelity, monitoring, sustainability, safety, and equity may be less developed than efficacy evidence.	Identify implementation and pragmatic studies; code reach, fidelity, reassessment, referral, safety, sustainability, differential access, and distribution of benefits and burdens.

CMDAS item	Preliminary concern to test	Evidence required before scoring
8. Portfolio alignment	Publication and funding activity may favor the established endpoint over the highest-value residual uncertainty.	Link publications to funding where possible, compare resource allocation across stages, and use transparent stakeholder priority-setting. Mark the item "not assessable" when funding data are unavailable.

G. Provisional Dual-Drift Interpretation

Claim-level finding: The selected synthetic claim produces a descriptive EDAS profile of 13/16, with concerns concentrated in inferential boundary crossing and implementation overreach.

Trajectory-level finding: CMDAS cannot be formally scored from the selective sources used in this worked example. The item map generates testable hypotheses—particularly around endpoint confinement and implementation deferral—but does not establish that the SDF research field has Causal Maturation Drift.

Appropriate conclusion

Do not classify the SDF literature as "Dual Drift" from this appendix. The defensible conclusion is: "The selected claim shows Evidence Drift concerns, while possible Causal Maturation Drift remains a trajectory-level hypothesis requiring systematic mapping."

H. Inferentially Faithful Corrective Actions

H.1. Claim and communication

A calibrated formulation is:

> *"Silver diamine fluoride can arrest some cavitated caries lesions in children over short-term follow-up, although certainty and applicability vary across comparisons and clinical contexts. It should be used as part of a diagnosis-led, monitored care pathway. Current lesion-arrest evidence does not by itself establish restoration substitution, completion of comprehensive care, long-term comparative effectiveness, or equitable implementation [31–33]."*

H.2. Research portfolio

A formal priority-setting process should examine whether a greater share of future research should address:

- long-term lesion status, recurrence, symptoms, pulpal outcomes, adverse events, and durability;
- comparative effectiveness against clinically relevant alternatives;
- child- and family-important outcomes, including function, esthetics, acceptability, burden, and quality of life;
- completion of, or transition to, definitive care within a monitored pathway;
- implementation fidelity, training, reassessment, referral, sustainability, and safety; and
- equity, including who receives SDF, who receives alternatives, and whether temporary disease control becomes a durable substitute for comprehensive care.

H.3. Guidelines and policy

- Separate evidence for lesion arrest from evidence for comprehensive care, treatment substitution, access, or equity.
- Specify diagnosis, informed consent, indications, monitoring, reassessment, referral, and exit or escalation criteria.
- Avoid treating program reach or uptake as proof of equitable benefit.

- State the missing bridge evidence and update recommendations as long-term and implementation data accumulate.

I. Minimum Methods for a Formal CMDAS Assessment

1. Publish or register a protocol defining the decision, trajectory, time window, eligibility criteria, and search strategy.
2. Create a reproducible publication set and code design, population, comparator, endpoint, follow-up, setting, and delivery model.
3. State why the initial signal is sufficiently characterized for the intended decision, using systematic review, cumulative evidence, or transparent expert consensus.
4. Define the principal residual uncertainty with input from clinicians, causal and implementation methodologists, caregivers, and youth representatives.
5. Use at least two independent assessors, report item-level agreement, and adjudicate disagreements.
6. Conduct sensitivity analyses for the selected time window, inclusion criteria, and assumptions about signal maturity.
7. Map funding and portfolio allocation only when reliable data are available; otherwise mark the relevant item not assessable.
8. Report raw item profiles and narrative uncertainty. Do not use "high/low" categories or rank researchers, journals, or disciplines until the instrument is validated.

J. Safeguards Against Misinterpretation

The worked example does not imply:

- that individual SDF studies are methodologically flawed;
- that SDF is ineffective for lesion arrest or lacks a legitimate role in selected cases;
- that researchers, journals, funders, or guideline panels acted negligently;
- that every additional arrest study is unnecessary; replication, subgroup evaluation, and context-specific research may be justified; or
- that numerical scores have validated diagnostic thresholds.

The intended use is constructive: to calibrate claims, make residual uncertainty explicit, and identify the evidence needed before broader clinical or policy conclusions are advanced.

K. Summary of the Revised Worked Example

Table S6. Final outputs

Component	Output	Permitted interpretation
EDAS target claim	13/16 (81.25%), descriptive only	Multiple claim-level concerns are present; no categorical label is assigned.
EDAS contrast claims	0/16, 4/16, and 13/16	The synthetic set demonstrates gradation and supports future cognitive and reliability testing.
CMDAS trajectory	Not scored	Formal scoring requires systematic trajectory mapping and independent appraisal.
Dual-Drift profile	Not assigned	Evidence Drift concerns are demonstrated for the selected claim; Causal Maturation Drift remains a testable hypothesis.
Recommended use	Supplementary worked example	Operationalizes the framework without overstating the evidence or validity of the instruments.

This worked example is illustrative and does not constitute a formal systematic assessment of the SDF literature. Formal CMDAS application requires explicit trajectory mapping and should not be inferred from a single claim, a selective set of publications, or the perception that many similar papers exist.

References Cited in This Supplement

31. Worthington HV, Lewis SR, Glenny AM, Huang SS, Innes NPT, O'Malley L, et al. Topical silver diamine fluoride (SDF) for preventing and managing dental caries in children and adults. Cochrane Database Syst Rev. 2024;11(11):CD012718. doi:10.1002/14651858.CD012718.pub2.

32. Crystal YO, Marghalani AA, Ureles SD, Wright JT, Sulyanto R, Divaris K, et al. Use of silver diamine fluoride for dental caries management in children and adolescents, including those with special health care needs. Pediatr Dent. 2017;39(5):E135–E145.

33. Baghdadi ZD. Equity or two-tier care? Guardrails for silver diamine fluoride and delegated early childhood caries pathways. Children. 2026;13(3):386. doi:10.3390/children13030386.

Supplementary Material S2

Application of Provisional Heuristic Thresholds for the Causal Maturation Drift Assessment Scale (CMDAS)

A. Purpose and Scope This supplement provides operational guidance and a worked example that illustrates how to apply provisional heuristic thresholds for the Causal Maturation Drift Assessment Scale (CMDAS). While the Evidence Drift Assessment Scale (EDAS) evaluates individual claims, the CMDAS assesses whether a defined literature trajectory continues to accumulate within a familiar domain while the highest-value residual uncertainty remains unresolved.

Determining when a field transitions from "disciplined progression" to "developmental under-resolution" requires clear procedures. Without explicit criteria, judgments about trajectory maturation risk becoming highly subjective. Therefore, this supplement introduces provisional numerical heuristics—specifically addressing study counts, publication proportions, and time windows—to operationalize the CMDAS items.

Interpretive Rule: The heuristics presented here are provisional proposals intended solely to make appraisal assumptions explicit and to facilitate future empirical testing. They are not validated thresholds, consensus standards, or formal decision rules. They should not be used to definitively classify research fields, evaluate investigators, reallocate funding, or support high-stakes policy decisions.

B. Provisional Heuristic Rules for CMDAS Scoring To score the CMDAS items, assessors must distinguish among "balanced progression" (Score 0), "emerging concentration" (Score 1), and "persistent concentration" (Score 2). The following rules are proposed for testing:

- **The Proportion Rule:** Over the most recent 3–5 years of a defined trajectory, if >60% of publications addressing the core question use the same design, exposure, and outcome without introducing a new inferential bridge, it indicates persistent concentration (Score 2). If the proportion is 30–60%, it indicates emerging concentration (Score 1). If <30%, it indicates balanced progression (Score 0).
- **The Time Rule:** If the same major evidentiary limitation (e.g., lack of longitudinal follow-up) has been explicitly acknowledged in the discussion sections of at least three consecutive review articles or guideline updates spanning more than three years, and no corresponding increase in studies addressing that limitation has occurred, the relevant items warrant a Score 2.
- **The Opportunity Rule:** If feasible methods to address residual uncertainty exist and have been successfully applied in analogous fields but remain absent from the target trajectory despite repeated calls for them, the score should be 2. If methods are not yet feasible, ethically constrained, or require substantial infrastructure, the score should be 1 or 0, with documented justification.

C. Worked Threshold Example: Salivary miRNA Biomarkers The following hypothetical scenario illustrates how an assessor would apply the CMDAS eligibility gate and heuristic rules to a mapped research portfolio.

1. Trajectory Definition and Evidence Mapping

- **Target Field:** A ten-year research portfolio focused on salivary miRNA biomarkers for the early detection of oral squamous cell carcinoma.
- **Current Portfolio Composition:**

    - 45 cross-sectional case–control studies (focused on discovery and internal validation).
    - 2 prospective cohort studies (limited by small sample sizes and single-center execution).
    - 0 studies examining clinical utility (i.e., whether the screening actually alters the stage at diagnosis, treatment pathways, or patient survival).
- **Expert Consensus/Reviews:** Systematic reviews published over the past four years have repeatedly stated: *"Prospective, population-based validation and clinical utility studies are urgently needed."*

2. Threshold Application and Scoring

- **Eligibility Gate (Signal Characterization):** Across the 45 cross-sectional studies, pooled Area Under the Curve (AUC) estimates are stable across 10+ validation cohorts (AUC ~0.85, with a narrow confidence interval). The signal is therefore considered strongly associated. The core residual uncertainty—prospective clinical impact—is clearly stated and remains unresolved. The trajectory passes the eligibility gate.
- **Applying the Proportion Rule (CMDAS Item 1: Same-domain accumulation):** Because >70% of the recent portfolio remains cross-sectional despite a stable AUC, this triggers the >60% heuristic. **Result: Score 2.**
- **Applying the Opportunity Rule (CMDAS Item 7: Implementation deferral):** Clinical utility and workflow integration studies are entirely feasible (population-based screening trials for biomarkers in other cancers, such as breast and prostate, exist) but remain absent from this specific portfolio. **Result: Score 2.**

Under this provisional heuristic approach, this pattern would yield scores of 2 across multiple CMDAS items, raising a structured concern about possible persistent Causal Maturation Drift.

D. Handling Ambiguous Cases When heuristic thresholds are not clearly met or constraints are highly context-dependent, assessors should:

1. Score conservatively (i.e., default to 0 or 1) and document the ambiguity in a narrative annex.

2. Seek a second independent score, especially for high-stakes applications (e.g., funding portfolio reallocation or systematic reviews).

3. Prioritize the item-level profile over the total score as the primary appraisal output. A provisional score of 2 on a potentially consequential item—for example, a persistent absence of temporal evidence—should prompt closer examination and narrative justification rather than an automatic classification or corrective action.

E. Summary All values and examples in this supplement are provisional proposals for future testing. No empirical evidence currently supports their use as validated diagnostic thresholds. Subsequent empirical studies must determine whether these numerical criteria are appropriate, whether they should be calibrated differently across diverse research contexts, and whether narrative decision principles ultimately prove more reliable than fixed percentages, study counts, or time windows.
